# Body adiposity index in assessing the risk of type 2 diabetes mellitus development: the Baependi Heart Study

**DOI:** 10.1186/s13098-019-0467-1

**Published:** 2019-08-29

**Authors:** Camila Maciel de Oliveira, Jessica Pavani, José Eduardo Krieger, Rafael de Oliveira Alvim, Carlos Alberto Mourão-Junior, Alexandre da Costa Pereira

**Affiliations:** 1 Laboratory of Genetics and Molecular Cardiology, Heart Institute (InCor), University of São Paulo Medical School, São Paulo, Brazil; 2 Department of Integrative Medicine, Federal University of Paraná, Curitiba, Brazil; 3 Department of Statistics, Pontificia Universidad Católica de Chile, Santiago, Chile; 4 Department of Physiological Sciences, Federal University of Amazonas, Manaus, Brazil; 5 Department of Physiology, Federal University of Juiz de Fora, Juiz de Fora, Brazil

**Keywords:** Baependi heart study cohort, Body adiposity index, Brazil, Type 2 diabetes mellitus

## Abstract

**Background:**

The association between diabetes and obesity is very well established. Faced with this, several anthropometric indices of adiposity are often involved in studies on diabetes. Our main goal in this paper is to evaluate the association between body adiposity index (BAI) and type 2 diabetes mellitus (T2DM) in a sample of the Brazilian population after 5-year follow-up.

**Methods:**

The data used come from the Baependi Heart Study cohort, which consists of two periods: cycle 1 (2005–2006) and cycle 2 (2010–2013). Individuals of both sexes (n = 1121) were selected by excluding participants with type 2 diabetes mellitus at baseline or those that were lost to follow-up.

**Results:**

The diabetic subjects showed higher systolic blood pressure, BAI, body mass index, waist circumference and fasting glucose levels. In addition, using mixed-effects logistic regression, we found that the elevation of a single unit of BAI represented an increase of 8.4% in the risk of a patient developing T2DM (OR = 1.084 [95% CI 1.045–1.124]).

**Conclusions:**

Obesity is recognised as one of the most important risk factors for T2DM and BAI has proven to be a useful tool in estimating the risk of a patient developing T2DM in a Brazilian population.

## Introduction

Diabetes Mellitus is a multifactorial metabolic disease associated with various conditions, including genetic predisposition, physical inactivity and mainly obesity. In fact, obesity is the second most important risk factor for various diseases [1] and its association with diabetes is very well established [2, 3]. As in the rest of the world, obesity has attracted attention in Brazil. More than half of the Brazilian population is overweight and almost 20% is obese [1]. The increasing incidence of obesity and increase in the incidence of type 2 diabetes (T2DM) has caused great concern about public policy.

Many studies have been carried out in this area, however, many gaps still need to be bridged. Previous studies have commonly discussed the effectiveness of anthropometric indices of adiposity in association with some cardiovascular risk factors [4]. Of these, the simplest ones, such as body mass index (BMI) and waist circumference (WC), have been most widely explored by the scientific community, although their limitations are well understood as applied to the prediction of pathologies such as T2DM in different populations. As an alternative, other parameters have been proposed, such as the body adiposity index (BAI), which take account simple measures such as hip circumference and height. This parameter, developed by Bergman et al. [5], has proven to be effective when related to some metabolic diseases.

Comparisons between BAI and other adiposity indexes have been also explored [6, 7]. However, according to Alvim et al. [2], ethnic differences may influence the discriminatory power of BAI, as well as several other anthropometric indices, in assessing the risk of T2DM. In agreement with this reality, our main interest is to investigate the influence of BAI on T2DM in a sample of the Brazilian population, after 5-year follow-up.

## Methods

The Baependi Heart Study cohort is a genetic epidemiological follow-up study of cardiovascular risk factors [8]. For the data collection process, individuals of both genders and aged 18–102 years were randomly considered, all from the municipality of Baependi. After recruitment, all relatives of the participants were invited to participate. Once selected, a physical examination was carried out and blood samples were collected. Currently, the cohort study consists of two periods: cycle 1 (2005–2006) and cycle 2 (2010–2013). The present study was approved by the ethics committee of the Hospital das Clínicas, University of São Paulo, Brazil (SDC: 3485/10/ 074), and each participant provided written informed consent before participation.

Based on this cohort study, 1225 individuals were selected following some inclusion and non-inclusion criteria. Initially, all the participants answered a questionnaire in which they stated whether they were affected by some type of diabetes or if they were users of some antidiabetic medication. If such questions had affirmative answers, the patient was not included in the study. After responding to the questionnaire, regardless of responses, patients were referred for blood screening. After screening test, T2DM was diagnosed by the presence of fasting plasma glucose ≥ 126 mg/dL or antidiabetic drug use [9]. In such case, the subject was not included in the cycle 1. Therefore, in cycle 1 we assessed only non-diabetic patients, accordingly to the criteria above (questionnaire and fasting plasma glucose). Five years after cycle 1, cycle 2 of the study was carried out.

In cycle 2 the patients in cycle 1 were reassessed and divided into two groups: (i) those who remained non-diabetic (Diabetes Free group) and (ii) those who had fasting plasma glucose levels greater than 126 mg/dL or reported use of antidiabetic drug (Incident Diabetes group).

In both cycles, blood triglycerides, total cholesterol, HDL-cholesterol, LDL-cholesterol and fasting glucose were evaluated by standard techniques in 12-h fasting blood samples [10].

Dyslipidaemia treatment was defined as percentage of individuals who used at least one class of lipid-lowering drug.

Anthropometric parameters were measured according to a standard protocol [10]. Height was measured in centimetres and weight in kilograms using a calibrated digital balance. WC was measured at the mean point between the lowest rib margin and the iliac crest with the subject standing and at the maximum point of normal expiration. Hip circumference was measured to the nearest 0.1 cm around the thighs, at the height of the greater trochanter, in the standing position. Increased WC was defined as ≥ 88 cm for women and ≥ 102 cm for men. The calculation of BAI was based on hip circumference and height (BAI = [hip circumference (cm)/(height (m) ^1.5^] – 18) [5].

Also in both cycles, blood pressure was measured using a standard digital sphygmomanometer (OMRON, Brazil) on the left arm after 5 min rest, in the sitting position. Systolic (SBP) and diastolic blood pressures (DBP) were calculated from three readings (mean value of all measurements), with a minimal interval of 3 min [10]. Hypertension was defined as mean SBP ≥ 140 mmHg and/or DBP ≥ 90 mmHg and/or antihypertensive drug use.

Clinical characteristics of patients in both cycles were assessed using descriptive statistics. Continuous variables were expressed as the mean ± SD and categorical variables were expressed as percentages. Normality of all data was tested with the Kolmogorov–Smirnov test. Mixed-effects logistic regression was used in order to verify the association between BAI and the incidence of T2DM, and examine how much risk it represents for the development of this chronic disease. The regression model was carried out having BAI as the main parameter, and sex, age, SBP, triglycerides and HDL-cholesterol as control variables. Taking into account the kinship relations among patients, we also considered family as an effect. All statistical analyses were carried out using the R (version 3.5.1) statistical software [11] with the level of significance set at 5%.

## Results

**Fig. 1:**
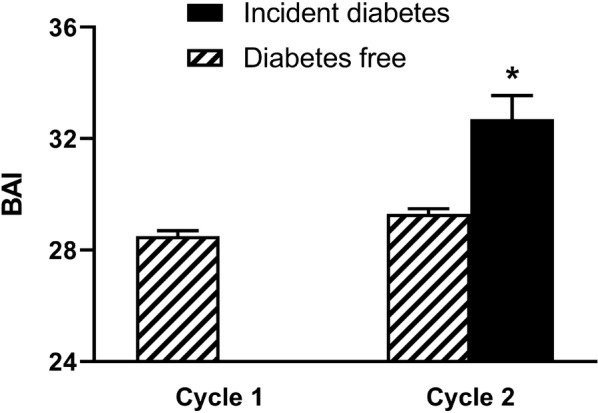
Graph showing BAI in cycle 1 and in two groups of cycle 2. Data are mean and SEM. The increase of BAI in Incident Diabetes group was statistically significant (p < 0.001)

Clinical, demographic, anthropometric, and biochemical data are summarised in Table 1. The percentage of men, hypertensives, obese (increased WC) and individuals treated with lipid-lowering drugs were higher in the group that became diabetic after 5-year follow-up. In addition, these individuals showed higher SBP, BMI, WC and fasting glucose levels. BAI increased only 3% between cycles 1 and 2. However, BAI was much higher in diabetics than non-diabetics in the cycle 2 (Fig. 1). In the present study, the incidence of T2DM after 5-year follow-up was 6.7% (75/1121).

**Table 1:** Characteristics of subjects in the sample

Variables	Cycle 1	Cycle 2		
		Diabetes free	Incident diabetes	*p*-value
n	1121	1046	75	–
Age, years	42.1 ± 16.1	46.9 ± 15.9	49.3 ± 16.6	0.24
Sex (% men)	44	43	47	< 0.001
Hypertension (%)	30	37	75	< 0.001
Increased WC (%)	30	42	61	< 0.001
Dyslipidaemia treatment (%)	3	7	24	< 0.001
SBP, mmHg	125.0 ± 18.5	125.2 ± 16.4	133.4 ± 17.8	< 0.001
DBP, mmHg	78.2 ± 11.2	76.7 ± 10.6	76.9 ± 10.3	0.88
BMI, kg/m2	24.3 ± 4.7	25.64 ± 4.9	28.45 ± 5.9	< 0.001
WC, cm	86.6 ± 11.79	90.6 ± 11.8	99.1 ± 11.6	< 0.001
Fasting glucose, mg/dL	87.5 ± 16.7	89.3 ± 10.2	137.3 ± 49.7	< 0.001
Total cholesterol, mg/dL	178.1 ± 46.9	200.6 ± 67.2	206.7 ± 51.9	0.33
HDL-cholesterol, mg/dL	56.4 ± 15.7	47.5 ± 11.8	46.2 ± 11.2	0.33
LDL-cholesterol, mg/dL	96.4 ± 42.8	125.3 ± 34.8	121.6 ± 44.9	0.51
Triglycerides, mg/dL	128.9 ± 68.9	123.8 ± 94.8	115.7 ± 78.6	0.39

Hypertension: systolic blood pressure ≥ 140 mmHg, diastolic blood pressure ≥ 90 mmHg and/or anti-hypertensive drug use. Dyslipidaemia treatment: percentage of individuals who used at least one class of lipid-lowering drug. Increased WC: ≥ 88 cm for women and ≥ 102 cm for men. Continuous data are expressed as the mean ± standard deviation and categorical data are expressed as percentage

SBP, systolic blood pressure; DBP, diastolic blood pressure; BAI, body adiposity index; BMI, body mass index; WC, waist circumference

Using mixed-effects logistic regression we found that BAI was significantly different between groups (p-value < 0.001). The main finding of our study was that the elevation of a single unit of BAI represented an increase of 8.4% in the risk of patient developing T2DM (odds ratio [OR] = 1.084 [95% CI 1.045–1.124]) **(**Table 2). This model was adjusted for SBP, sex, age, triglycerides and HDL-cholesterol.

**Table 2:** Variables associated with diabetes mellitus in a logistic regression analysis

Variables	OR	95% CI	*p*-value
BAI	1.084	1.045–1.124	* < 0.001*
Sex	1.306	0.764–2.234	0.329
Age	1.004	0.988–1.020	0.633
SBP	1.025	1.011–1.040	*< 0.001*
Triglycerides	1.000	0.997–1.003	0.956
HDL	0.994	0.972–1.016	0.597

Diabetes mellitus: diagnosis was established in patients with fasting glucose equal to or greater than 126 mg/dL, or in patients who were under the use of anti-diabetic medicine

Predictive variable: BAI

Control variables: sex, age, PAS, triglycerides and HDL

BAI, body adiposity index; SBP, systolic blood pressure; HDL, high-density lipoprotein cholesterol

## Discussion

The main finding of our study was the association between BAI and T2DM in a Brazilian population in which the elevation of a single unit of BAI represented an increase of 8.4% in the risk of patient developing T2DM, even after adjusting for confounding variables such as age, sex, triglycerides, HDL-cholesterol and SBP.

Several studies have focused on the relationship between anthropometric indices of adiposity and diabetes mellitus in samples from different populations. In Brazil, Flor et al. [3] and Freitas [12] demonstrated a strong association between T2DM and obesity, considering different Brazilian population samples. Supporting such studies, our findings confirm this relationship, highlighting BAI as an effective parameter. Corroborating with the findings of Bergman et al. [5] and López et al. [7], our study highlights that an increasing BAI implies a significant increase in the risk of developing T2DM. Therefore, BAI is a relevant tool to predict T2DM risk in the Baependi population.

This study has some limitations. First, all the participants live in Baependi, a small town with great rural activity located in South-eastern Brazil. Therefore, these results cannot be extrapolated to the general Brazilian population. Second, because it is an observational study where the participant makes a single visit per cycle, it becomes impracticable to diagnose diabetes through two measures of fasting glycaemia on different days. Therefore, like other observational studies, the diagnosis of diabetes is based on a single measurement of fasting glucose (≥ 126 mg/dL) or if the participant reports the use of hypoglycaemic drugs. On the other hand the main strengths are that our study has an appropriate sample size, which allowed for high statistical power, and, additionally, the anthropometric measures were obtained by a single investigator.

In conclusion, obesity is recognised as one of the most important risk factors for T2DM. The results of our study showed that BAI could be a useful tool for the assessment of T2DM risk in a Brazilian population.

## Data Availability

The data sets used and/or analysed during the current study are available from the corresponding author on reasonable request.

## Abbreviations

BMI: body mass index
BAI: body adiposity index
WC: waist circumference
OR: odds ratio
T2DM: type 2 diabetes mellitus
PAS: systolic blood pressure
DBP: diastolic blood pressure
